# Absence of unidirectionally propagating surface plasmon-polaritons at nonreciprocal metal-dielectric interfaces

**DOI:** 10.1038/s41467-020-14504-9

**Published:** 2020-02-03

**Authors:** Siddharth Buddhiraju, Yu Shi, Alex Song, Casey Wojcik, Momchil Minkov, Ian A. D. Williamson, Avik Dutt, Shanhui Fan

**Affiliations:** Ginzton Laboratory, Department of Electrical Engineering, Stanford University, Stanford, CA USA

**Keywords:** Magneto-optics, Nanophotonics and plasmonics

## Abstract

In the presence of an external magnetic field, the surface plasmon polariton that exists at the metal-dielectric interface is believed to support a unidirectional frequency range near the surface plasmon frequency, where the surface plasmon polariton propagates along one but not the opposite direction. Recent works have pointed to some of the paradoxical consequences of such a unidirectional range, including in particular the violation of the time-bandwidth product constraint that should otherwise apply in general in static systems. Here we show that such a unidirectional frequency range is nonphysical using both a general thermodynamic argument and a detailed calculation based on a nonlocal hydrodynamic Drude model for the metal permittivity. Our calculation reveals that the surface plasmon-polariton at metal-dielectric interfaces remains bidirectional for all frequencies.

## Abstract (web summary)

The local Drude model predicts that, under certain conditions, surface plasmon polaritons at a metal-dielectric surface have a frequency range where only unidirectional propagation is supported. Here, the authors show that in more realistic non-local models surface plasmon polaritons exhibit bidirectional propagation for all frequencies.

## Introduction

In the past 2 decades, there have been significant developments in the field of plasmonics, which explores surface-plasmon polaritons that exist at metal–dielectric interfaces to achieve nanoscale control of light^1–3^. Most plasmonic structures satisfy the Lorentz reciprocity theorem^4^. On the other hand, in the presence of an external magnetic field, the behavior of surface-plasmon polaritons becomes nonreciprocal. Such nonreciprocal surface-plasmon polaritons have generated substantial interest^5–8^ since they represent a fundamentally different regime of light propagation, having potential importance for applications such as sensing and information processing.

A particularly significant effect of nonreciprocal plasmons is the existence of a unidirectional frequency range. Such unidirectional frequency ranges have been shown to occur in topologically nontrivial metal–metal interfaces^9–14^, where the unidirectional behavior is linked to the topology of the bandstructure, or in metal–dielectric systems^15–18^. In this paper, we will discuss metal–dielectric systems. With the metal described by the Drude model, in the presence of an external magnetic field, there exists a frequency range where the surface-plasmon polariton can only propagate along one direction. In a recent paper^18^, it was noted that the existence of such unidirectional surface-plasmon polaritons can lead to resonator structures that violate the time-bandwidth product constraint that should otherwise apply in general in static systems^19^. Subsequently, refs. ^20,21^ argued that breaking the time-bandwidth product should not be possible based on a coupled-mode theory analysis, and that doing so may violate the second law of thermodynamics. Motivated by these considerations, it becomes important to re-examine the fundamental physical assumptions that give rise to unidirectionally propagating surface-plasmon polaritons at metal–dielectric interfaces. Within the local Drude model, the existence of the unidirectional frequency range depends on the behavior of the model in the limit of large wavevectors. On the other hand, it has long been known^22^ that nonlocal effects become important in this limit.

In this paper, we show that there should not be a unidirectional frequency range in the spectrum of the surface-plasmon polariton at metal–dielectric interfaces once nonlocal effects are considered. Instead, there will always be propagating modes in both directions. We illustrate this by detailed calculations based on the hydrodynamic model for the metal dielectric function. We also present a general thermodynamic argument to show that the main conclusion of the paper, i.e., the absence of unidirectionally propagating surface-plasmons polaritons at nonreciprocal metal–dielectric interfaces, should hold for any physical nonlocal model of the dielectric function.

## Results

### Drude model

We start with a brief review of the dispersion relation of the surface-plasmon polariton at the metal–dielectric interface. Throughout the paper, for simplicity, we refer to any system with a strong plasmonic response as a metal. In addition to the usual free-electron metals, such “metals” also include heavily doped semiconductors that exhibit a plasmonic response at infrared wavelengths. Within the local Drude model, in the presence of a static magnetic field ${{\bf{B}}}_{0}=-{B}_{0}\hat{{\bf{y}}}$, the frequency(*ω*)-dependent dielectric function of metal has the following form:

1 $$\frac{{\epsilon }_{{{m}}}(\omega )}{{\epsilon }_{\infty }}= \, 	1-\frac{{\omega }_{{{p}}}^{2}}{{(\omega +i{\gamma }_{0})}^{2}-{\omega }_{{{c}}}^{2}}\\ 	 \, \times \left(\begin{array}{rcl}1+i\frac{{\gamma }_{0}}{\omega }&0&i\frac{{\omega }_{{{c}}}}{\omega }\\ 0 &\frac{{(\omega \, +\, i{\gamma }_{0})}^{2}\, -\, {\omega }_{{{c}}}^{2}}{\omega (\omega \, +\, i{\gamma }_{0})}&0\\ -i\frac{{\omega }_{{{c}}}}{\omega }&0&1+i\frac{{\gamma }_{0}}{\omega }\end{array}\right),$$

where *ϵ*_*∞*_ is the dielectric response from the bound electrons and ions, *ω*_*p*_ the plasma frequency, *γ*_0_ the phenomenological scattering loss rate, and *ω*_*c*_ = *eB*_0_  ∕ *m* the cyclotron frequency, with fundamental charge *e* and effective mass of the free carriers *m*.

**Fig. 1:**
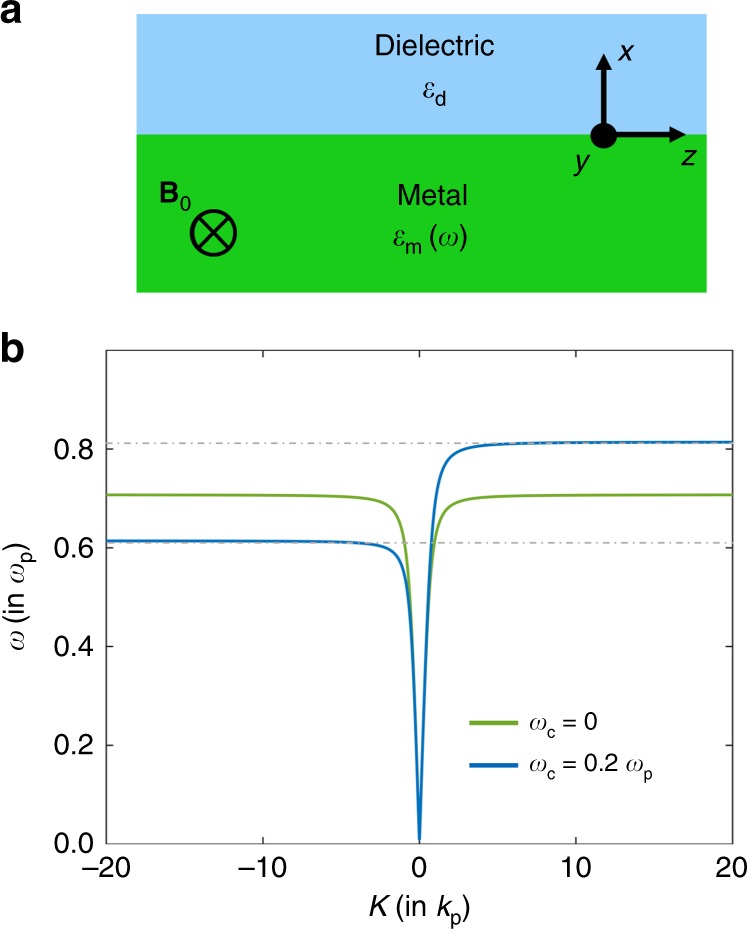
Surface-plasmon-polariton dispersion relation in the local Drude model. **a** An interface between a dielectric and a metal described by the Drude model. **b** Dispersion relation of surface-plasmon-polariton propagation at the interface in the absence (green curve) and in the presence (blue curve) of an external magnetic field **B**_0_ as indicated in **a**. *K* is the wavevector component parallel to the interface, *k*_*p*_ = *ω*_*p*_*/c*, and *ω*_*c*_ = *eB*_0_∕*m* is the cyclotron frequency.

Consider the metal–dielectric interface shown in Fig. 1a. When *B*_0_ = 0, in the near-lossless limit of *γ*_0_ → 0, the dispersion relation *ω*(*K*) of the surface-plasmon polariton is shown by the green curve in Fig. 1b, where *K* is the wavevector parallel to the interface. Here we assume that *ϵ*_*∞*_ = 1, and that the dielectric is air with *ϵ*_*d*_ = 1. Since the system is reciprocal, we have *ω*(*K*) = *ω*(−*K*). In the *K* → ±*∞* limit, the frequency of the surface-plasmon-polariton approaches the surface-plasmon frequency ${\omega }_{{\rm{sp}}}={\omega }_{{{p}}}/\sqrt{2}$.

When *B*_0_ ≠ 0, again assuming the near-lossless limit, the dispersion relation for the same interface system is shown by the blue curve in Fig. 1b. Since the system is no longer reciprocal, we have *ω*(*K*) ≠ *ω*(−*K*). Moreover, the surface-plasmon frequencies for the forward and backward directions are unequal at the ∣*K*∣ → *∞* limit, opening a unidirectional frequency range around *ω*_sp_ where the surface plasmon polariton propagates only along the positive-*K* direction. The existence of such a unidirectional frequency range is the key to the unusual time-bandwidth product behavior reported in ref. ^18^. On the other hand, as observed from Fig. 1b, the existence of such a unidirectional range is strongly dependent on the behavior of the Drude model in the ∣*K*∣ → *∞* limit. And yet, it is well known that the local Drude model of Eq. (1) is no longer adequate in this limit^22^, and instead the spatially dispersive or “nonlocal” behavior of the electromagnetic response of the metal must be considered. For example, it was also shown^11^ that topological effects in continuous media need to be described taking into account nonlocality. Therefore, to understand the potential physics of such unidirectional propagation, it is essential to consider the effect of the nonlocal dielectric function.

### Hydrodynamic Drude model

There exist many treatments of nonlocality, such as those based on the hydrodynamic model^23,24^, the random phase approximation^25,26^, and a quantum-corrected model^27^. The description of plasmonic properties using these models is closely related to the emerging area of quantum plasmonics, where the quantum nature of the electron gas plays a significant role^27–29^. Here, we briefly discuss the hydrodynamic model, a simple analytic model that has often been used to describe nonlocal response in deep subwavelength metallic structures^23,30^, and recently in nanoparticles made of doped semiconductors such as indium antimonide (InSb)^31^. We refer readers to refs. ^30,32^ and references therein for a detailed overview of nonlocality in surface-plasmon polaritons as well as a derivation of the hydrodynamic model.

In this model, the collective motion of electrons is described using a density *n*(**r**, *t*), a velocity **v**(**r**, *t*), and an energy functional that can be appropriately chosen to describe the internal kinetic energy as well as interactions. We follow ref. ^30^ to employ the Thomas–Fermi approximation for the energy functional. The equations of motion of the free carriers in this approximation are given by^33,34^

2 $$\frac{\partial {\bf{v}}}{\partial t}+\gamma {\bf{v}}+({\bf{v}}\cdot \nabla ){\bf{v}}=-\frac{e}{m}({\bf{E}}+{\bf{v}}\times {\bf{B}})-{\beta }^{2}\frac{\nabla n}{n},$$

3 $$\frac{\partial n}{\partial t}=-\nabla \cdot (n{\bf{v}}),$$

where *β* is the nonlocal parameter proportional to the Fermi velocity *v*_*F*_^35^,

4 $${\beta }^{2}=\frac{\frac{3}{5}\omega +\frac{1}{3}i\gamma }{\omega +i\gamma }{v}_{{{F}}}^{2}.$$

Linearizing Eqs. (2) and (3), and defining the free-electron current **J** = −*en*_0_**v**, where *n*_0_ is the equilibrium electron density, a single equation can be obtained for **J** in the frequency domain as

5 $${\beta }^{2}\nabla (\nabla \cdot {\bf{J}})+\omega (\omega +i\gamma ){\bf{J}}=-i\omega ({\omega }_{{{p}}}^{2}{\epsilon }_{\infty }{\epsilon }_{0}{\bf{E}}-\frac{e}{m}{\bf{J}}\times {{\bf{B}}}_{0}),$$

where **B**_0_ is an externally applied dc magnetic field. This equation is coupled with Maxwell’s equations, written using the **E** field as

6 $$\nabla \times \nabla \times {\bf{E}}=\frac{{\omega }^{2}}{{c}^{2}}{\epsilon }_{\infty }{\bf{E}}-i\omega {\mu }_{0}{\bf{J}}.$$

Unlike in the local model, the presence of the nonlocal term *β*^2^ ∇ (∇⋅) in this model requires an additional boundary condition^30^ to determine the dispersion relation. Here, the additional boundary condition required to solve Eqs. (5) and (6) is

7 $${\bf{J}}\cdot \hat{{\bf{n}}}=0,$$

where $\hat{{\bf{n}}}$ is the unit vector normal to the metal–dielectric interface. This has the effect of imposing an infinite potential well for the electron gas at the metal–dielectric boundary.

**Fig. 2:**
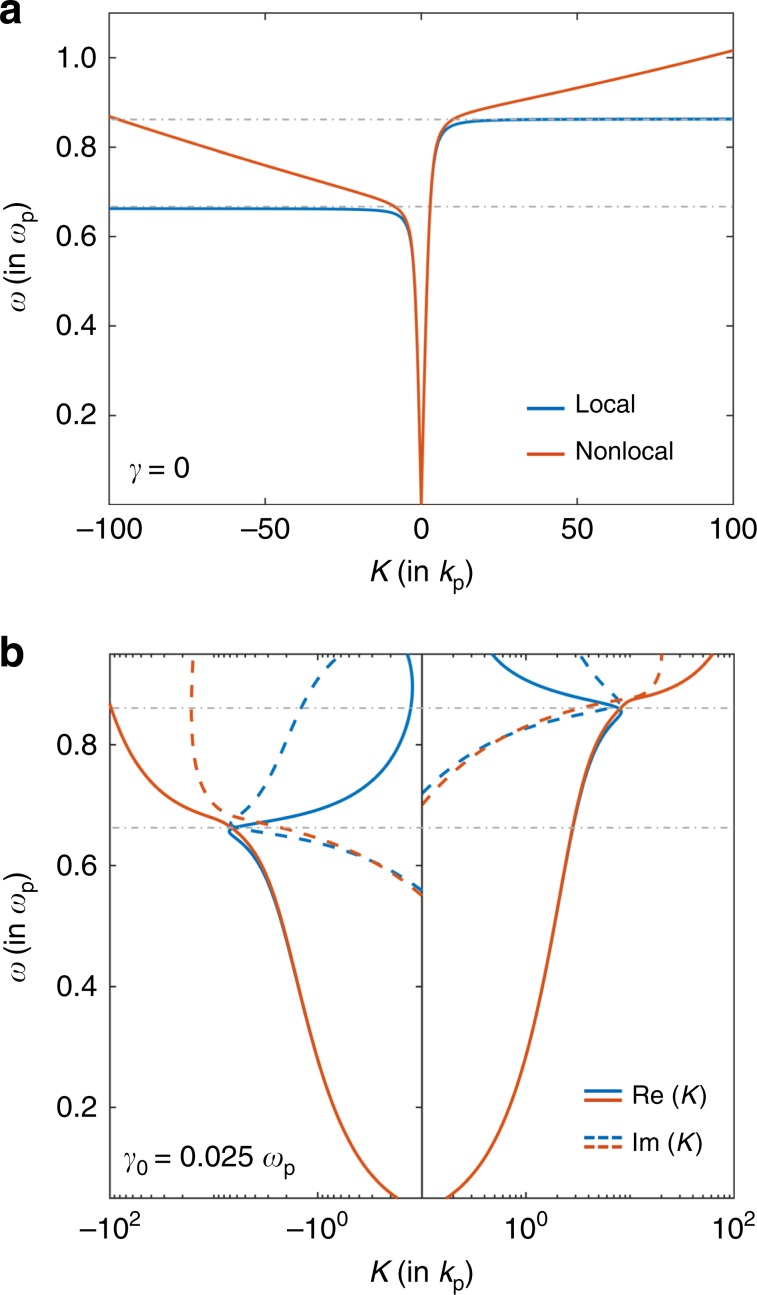
Dispersion relations in the local and hydrodynamic Drude models. Dispersion relation for the interface considered in ref. ^18^, in the local (blue) and nonlocal (red) models. **a** A flat dispersion relation is obtained in the lossless (*γ*_0_ = 0) local Drude model, resulting in a unidirectional gap indicated by the dotted gray lines. This gap is removed in the hydrodynamic Drude model with a high-*K* counter-propagating wave. **b** Real (solid line) and imaginary (dotted line) parts of *K* when *γ*_0_ = 0.025*ω*_*p*_ in the local (blue) and the nonlocal (red) models. Landau damping is self-consistently incorporated in the nonlocal model. ${\rm{Re}}(K)\,<\,{\rm{Im}}(K)$ in the unidirectional gap in the local model, while ${\rm{Re}}(K)\,> \,{\rm{Im}}(K)$ in the nonlocal model. Note that *K*∕*k*_*p*_ is in log scale.

To illustrate the effect of nonlocality on the nonreciprocal behavior of the surface-plasmon polaritons, we consider an interface where the dielectric layer is silicon (*ϵ*_*d*_ = 11.68) and the metallic layer is n-doped InSb, a material commonly used in demonstrating magneto-optical plasmonic effects^15,18^. This interface was previously used in ref. ^18^, with the InSb layer treated using the local Drude model. The InSb layer has *ϵ*_*∞*_ = 15.6 and plasma frequency *ω*_*p*_ = 2π × (2 × 10^12^ Hz). A constant dc magnetic field of *B*_0_ = 0.2 T is applied in the $-\hat{{\bf{y}}}$ direction to break reciprocity. Owing to a rather small conductivity effective mass for electrons, a large value^31^ of *β* = 1.07 × 10^6^ ms^−1^ is obtained at 300 K. Thus, the effect of nonlocality, which was not considered in ref. ^18^, is in fact prominent in the dielectric response of InSb. In order to highlight the difference between the hydrodynamic model and the local Drude model, we first set *γ*_0_ = 0. Using these parameters, we solve Eqs. (5) and (6) for surface-plasmon polariton dispersion relation at the Si–InSb interface. The red curve in Fig. 2a depicts the dispersion relation in the hydrodynamic model, and the blue curve in the local Drude model. The dispersion relations from the two models are almost the same for small *K*, but deviate as *K* becomes larger. In particular, within the hydrodynamic model, there is no longer a unidirectional frequency range. At every frequency, there are both a propagating and a counter-propagating mode. We also note that the predictions between the local and nonlocal models start to deviate for *K* = 0.4 μm^−1^. Thus, in this system, nonlocal effects become important even for surface-plasmon waves with wavelength on the submicron scale, in contrast with standard plasmonic metals where nonlocal effects are important only when the plasmon wavelength is at the nanoscale.

The qualitative difference between the hydrodynamic model and the local Drude model persists over a wide range of loss rates *γ* in Eq. (2). The loss in a surface-plasmon polariton arises not only from scattering, but also surface-induced Landau damping^36,37^. Unlike bulk plasmons where Landau damping occurs only for *K* > *ω*∕*v*_*F*_, surface modes also experience Landau damping at smaller wavevectors owing to confinement in the direction normal to the interface. Following ref. ^36^, we write *γ* = *γ*_0_ + *γ*_*s*_, where *γ*_0_ is the loss rate from scattering and

8 $${\gamma }_{{{s}}}\,=\,\frac{3{\rm{\pi }}\omega }{2}\frac{{\int }_{1}^{\infty }{q}^{-3}| {F}_{x}(q){| }^{2}{\rm{d}}q}{{\int }_{0}^{\infty }| {F}_{x}(q){| }^{2}\,+\,| {F}_{z}(q){| }^{2}{\rm{d}}q}$$

is the loss rate from Landau damping. Here, *F*_*x*,*z*_(*q*) is the Fourier transform of the electric field *E*_*x*,*z*_(*x*) in the metal, and *q* is normalized to the onset of Landau damping, i.e., to *ω*∕*v*_*F*_. Since *γ*_*s*_ depends on the field profiles which in turn depend on *γ*_*s*_, we solve for the damping rate and the fields in a self-consistent manner.

In Fig. 2b, we plot the dispersion relation for *γ*_0_ = 0.025*ω*_*p*_ in blue for the local model and red for the nonlocal model. In the nonlocal model, the effective loss rate is given by *γ*_0_ + *γ*_*s*_ described above. The solid lines represent ${\rm{Re}}(K)$, while the dotted lines represent ${\rm{Im}}(K)$. Within the local model, while propagation is not strictly unidirectional in the presence of loss, the counter-propagating mode is significantly overdamped (${\rm{Re}}(K)\,<\,{\rm{Im}}(K)$) in the unidirectional frequency range, marked by the gray dotted lines. On the other hand, the counter-propagating mode continues to remain underdamped (${\rm{Re}}(K)\,> \,{\rm{Im}}(K)$) in the nonlocal model. Only for substantially high values of loss (*γ*_0_ > 0.05*ω*_*p*_) does the dispersion relation in the nonlocal model return approximately to its local form, in which case damping is high enough that the propagation of the surface-plasmon polariton is no longer apparent. The analysis here indicates that the effect of nonlocality on nonreciprocal surface-plasmon polaritons should be pronounced for a wide range of values of loss.

**Fig. 3:**
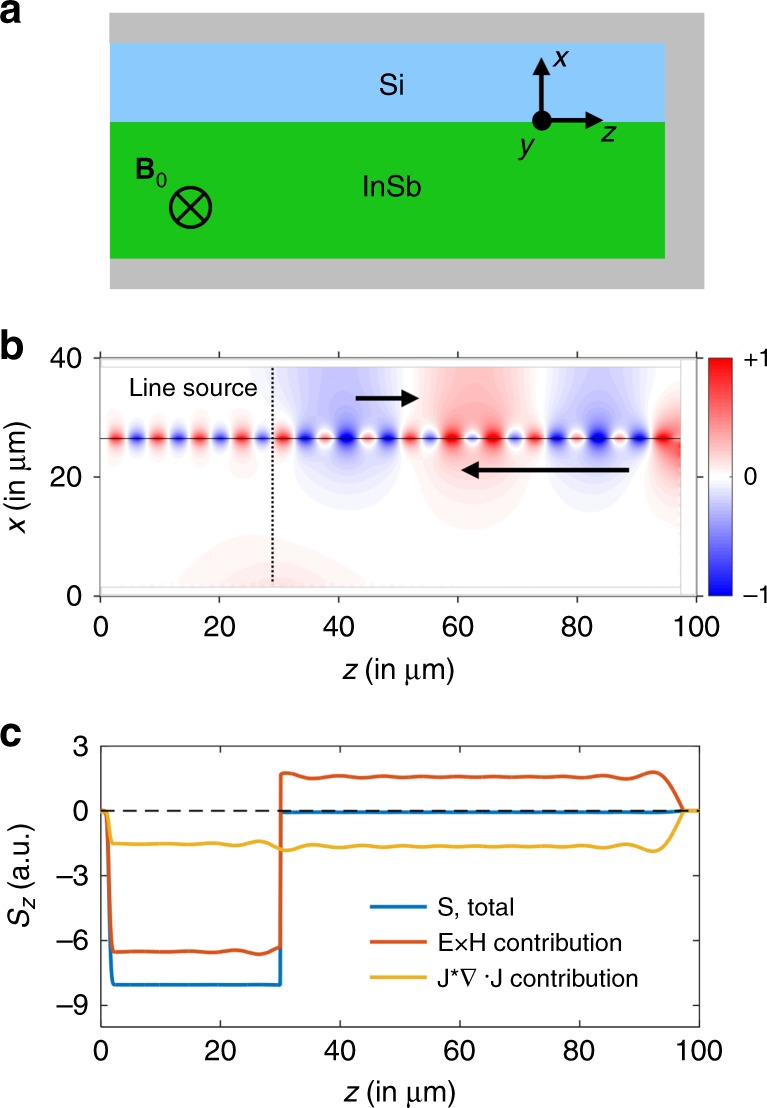
Numerical simulation of a truncated waveguide using the hydrodynamic model. A finite-difference frequency-domain (FDFD) algorithm is used to obtain the numerical results, at *ω* = 0.7*ω*_*p*_. **a** The structure as considered  in ref. ^18^. **b** Field profile of *H*_*z*_ generated by the indicated line source, clearly depicting a backward propagating mode with a significantly smaller wavevector than the forward propagating mode. **c** A finite Poynting flux in the backward direction relative to the line source, and a zero Poynting flux forward owing to the PEC to the right. The blue curve is the total Poynting vector from Eq. (9), while the red and yellow curve are its electromagnetic and kinetic terms, respectively, from Eq. (9).

In order to numerically demonstrate the effect of nonlocality on nonreciprocal photon transport, we re-examine the structure shown in Fig. 3a, which was first considered in ref. ^18^. The structure is two-dimensional and consists of the Si–InSb interface as discussed above, subject to a static out-of-plane magnetic field. Such an interface thus behaves as a nonreciprocal plasmonic waveguide. The waveguide is surrounded by a metal region, which serves both to truncate the waveguide at one end, as well as to eliminate any radiation losses. In ref. ^18^, the surrounding region was assumed to be silver. Here for simplicity we assume a surrounding region made of a perfect electric conductor (PEC), which makes very little difference to the simulations since the operating frequency, in the far-infrared region, is far below the plasma frequency of silver. Ref. ^18^ treats the InSb layer using the local dielectric function of Eq. (1). The choice of the magnetic field along the $-\hat{{\bf{y}}}$ direction results in a unidirectional frequency range where there is a surface-plasmon polariton propagating toward the truncation, but not in the opposite direction. Consequently, at a frequency inside the unidirectional range, ref. ^18^ shows that the electromagnetic field will propagate toward and be trapped at the truncation, with no leakage either in the backward direction, or through radiation losses. Such a trapping effect appears to lead to the violation of the time-bandwidth product constraint.

On the other hand, as we have discussed above, the nonlocal behavior is in fact intrinsic and rather significant in the dielectric response of InSb. Therefore, we extend the finite-difference frequency-domain method^38^ to include the nonlocal response as described by Eq. (5) for the InSb region, and resimulate the structure in Fig. 3a. To highlight the fact that there is a backward propagating mode even in the lossless system, we assume *γ* = 0. We excite the waveguide mode by placing a line source normal to the interface. We choose *ω* = 0.7*ω*_*p*_, a frequency that is inside the unidirectional range of the local model. In Fig. 3b, we plot the field distribution of *H*_*z*_, the *z*-component of the magnetic field of the excited surface-plasmon-polariton mode. We observe a significant excitation of backward propagating surface-plasmon polariton, as well as significant reflection at the truncation, in consistency with our dispersion relation analysis as shown in Fig. 2. In the Supplementary Materials, we provide movies to compare the field evolution in the local (Supplementary Movies 1 and 2) and nonlocal (Supplementary Movies 3 and 4) models in the presence of losses. Even in the presence of losses, a strong reflection into the high-*K* backward propagating mode is seen in the nonlocal model. On the other hand, no such effect of backward propagation is visible in the local model. These simulations indicate that the difference between the predictions of the local and nonlocal models are qualitatively different even in the presence of losses.

To further highlight the contrast between the local and nonlocal models, we note that, for *γ* = 0, the local Drude model would predict that within the unidirectional frequency range, there is a net energy flux toward the truncation, as ref. ^18^ shows. On the other hand, we compute the Poynting vector flux along the *z*-direction in the nonlocal model. The time-averaged Poynting vector **S** in the hydrodynamic model can be derived by combining the linearized forms of Eqs. (2) and (3) with the Poynting theorem to obtain

9 $${\bf{S}}=\frac{1}{2}{\rm{Re}}\left[{\bf{E}}\times {{\bf{H}}}^{* }+{\rm{i}}\frac{{\beta }^{2}}{\omega {\omega }_{{{p}}}^{2}{\epsilon }_{\infty }{\epsilon }_{0}}{{\bf{J}}}^{* }(\nabla \cdot {\bf{J}})\right].$$

We show the Poynting flux along the *z*-direction in Fig. 3c. The total Poynting flux (blue curve) within the hydrodynamic model contains contributions from both the electromagnetic field (**E** × H^*^, red curve) and the kinetic energy of the free carriers (${{\bf{J}}}^{* }\left(\nabla \cdot {\bf{J}}\right)$, yellow curve), unlike in the local model. Since there is a PEC termination, the total Poynting vector to the right of the source must be zero. We see that this is indeed the case, with the forward propagating electromagnetic component being canceled exactly by the counter-propagating kinetic component, an effect arising from the nonlocal term J^*^(∇ ⋅ **J**). Similarly, a negative value of Poynting flux is observed to the left of the source, also confirming the excitation of the high-*K* mode propagating backward.

### General thermodynamic argument

**Fig. 4:**
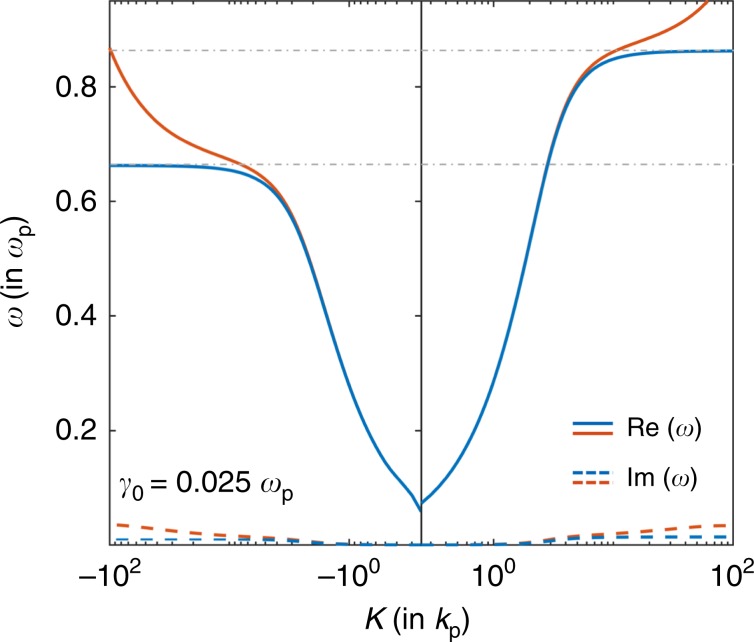
Dispersion relations in the real-wavevector complex-frequency picture. The dispersion relations in the real wavevector and complex-frequency picture for the surface-plasmon polariton in the local Drude model (blue) is contrasted with that in the hydrodynamic model (red). The solid and dotted lines represent the real and imaginary parts of the frequency, respectively, for a loss rate of *γ*_0_ = 0.025*ω*_*p*_. In the local model, the flat asymptote in the limit of *K* → *∞* persists even upon the inclusion of losses, resulting in an infinite number of states. By contrast, in the hydrodynamic model, the unidirectional frequency gap is closed in the real-wavevector complex-frequency picture as well as the complex-wavevector real-frequency picture (shown in Fig. 2b).

In the local Drude model, the surface plasmon has a dispersion relation *ω*(*K*) that asymptotically approaches a constant in the limit of *K* → *∞*, which results in an infinite number of states in a finite frequency range. From Fig. 1, this asymptotic behavior is apparent when the Drude model is lossless. However, this is also the case in the presence of losses in the Drude model: the dispersion relation relevant to computing the number of states in the presence of losses is obtained by setting a real wavevector and solving for a complex frequency^39^. It was shown^39,40^ that such a dispersion relation presents a flat asymptote at the surface-plasmon frequency even in the presence of losses. In Fig. 4, we plot the surface-plasmon-polariton dispersion relation in the presence of losses for a real wavevector and complex frequency for the local Drude model in blue, with the real part of the frequency shown by the solid curve and the imaginary part by the dotted curve. Note that for the local model, the flat asymptotes persist even in the presence of losses in the real-wavevector complex-frequency picture. In metal–dielectric systems, this asymptotic behavior is key to the existence of the unidirectional frequency range when a magnetic field is applied. However, since the asymptotic behavior also implies an infinite number of states in a finite frequency range, the thermal energy contained in the system diverges to infinity at any nonzero temperature. Any physical system should not have infinite thermal electromagnetic energy density at a finite temperature. Thus, the dispersion relation of the local Drude model and the resulting unidirectional frequency range are not physical. Further, our prediction that a unidirectional frequency range does not arise when a more realistic nonlocal model is used should therefore hold true independent of the details of the nonlocal model, since any valid nonlocal correction must remove the diverging number of states in the local Drude model and thereby remove the asymptotic behavior. As an example, the nonlocal hydrodynamic model considered in this paper indeed removes the flat asymptotic behavior in the real-wavevector complex-frequency picture, shown by the red curve in Fig. 4.

Related to the general thermodynamic argument above, it was shown^37^ that the leading order correction from quantum plasmonics to the surface-plasmon polariton in the local Drude model has a model-independent form of *ω* − *ω*_sp_ ~ *CK* for some constant *C*. Moreover, it was argued that the leading order nonlocal correction to the dynamics of the electron gas is ${\mathcal{O}}({K}^{2})$^41^ regardless of the microscopic model of nonlocality, with this correction also being the origin of the slope of the dispersion relation for large *K* in Fig. 2. These results concur with our observation above that the prediction of *ω* → *ω*_sp_ in the large-*K* limit from the local Drude model is unphysical, and thus any effect that relies upon such asymptotic behavior needs to be examined carefully.

## Discussion

In this paper, we show that the unidirectional frequency range, which is predicted for a metal–dielectric interface where the free-electron metal under a static magnetic field is described using a local Drude model, is nonphysical. We present a general argument from thermodynamic considerations and illustrate the argument with an explicit calculation using a more realistic nonlocal hydrodynamic model for the metal. Our results here suggest that the anomalous time-bandwidth product predicted by ref. ^18^, which arises as a direct consequence of the existence of the unidirectional frequency range, is not physical either. More generally, our work highlights the importance of using a more realistic permittivity model, such as those derived from quantum plasmonics considerations^27–29^, to understand nonreciprocal plasmonic effects.

## Methods

### Numerical simulation

The field and Poynting flux in Fig. 3b, c were obtained by solving Eqs. (5) and (6) using the finite-difference frequency-domain method^38^.

## Supplementary information

Description of Additional Supplementary Files

Supplementary Movie 1

Supplementary Movie 2

Supplementary Movie 3

Supplementary Movie 4

## Data Availability

The data that support the findings of this study are available from the corresponding author upon reasonable request.
