# Supplementary material for: Absence of unidirectionally propagating surface plasmon-polaritons at nonreciprocal metal-dielectric interfaces
Source: Nat Commun. 2020 Feb 3;11:674. doi: 10.1038/s41467-020-14504-9 (PMC6997186; doi:10.1038/s41467-020-14504-9)
Supplement: Supplementary file 1 — Description of Additional Supplementary Files [file 41467_2020_14504_MOESM1_ESM.pdf]

Description of Additional Supplementary files

Filename: Supplementary Video 1 (Simulation of the truncated waveguide in the local model for γ_0= 0.01ω_p)
Description: This movie shows the time evolution of the field in the truncated waveguide in the local model for γ_0= 0.01ω_p

Filename: Supplementary Video 2 (Simulation of the truncated waveguide in the local model for γ_0= 0.025ω_p)
Description: This movie shows the time evolution of the field in the truncated waveguide in the local model for γ_0= 0.025ω_p

Filename: Supplementary Video 3 (Simulation of the truncated waveguide in the nonlocal model for γ_0= 0.01ω_p)
Description: This movie shows the time evolution of the field in the truncated waveguide in the hydrodynamic model for γ_0= 0.01ω_p

Filename: Supplementary Video 4 (Simulation of the truncated waveguide in the nonlocal model for γ_0= 0.025ω_p)
Description: This movie shows the time evolution of the field in the truncated waveguide in the hydrodynamic model for γ_0= 0.025ω_p
